# Impact of Cold Storage Temperature and Shelf Life on Ripening Physiology, Quality Attributes, and Nutritional Value in Apricots—Implication of Cultivar

**DOI:** 10.3390/plants12152875

**Published:** 2023-08-04

**Authors:** Mina Kafkaletou, Anna Velliou, Miltiadis V. Christopoulos, Georgia Ouzounidou, Eleni Tsantili

**Affiliations:** 1 Laboratory of Pomology, Department of Crop Science, Agricultural University of Athens, Iera Odos 75, 11855 Athens, Greece; annavelliou2@gmail.com (A.V.); etsantili@aua.gr (E.T.); 2 Institute of Technology of Agricultural Products, Hellenic Agricultural Organization—DEMETER, S. Venizelou 1 Str., Lycovrissi, 14123 Athens, Greece; miltchrist@elgo.gr (M.V.C.); geouz@yahoo.gr (G.O.)

**Keywords:** *Prunus armeniaca* L., cold storage, shelf life, quality, chilling injuries, carotenoids, antioxidants

## Abstract

This work aimed to investigate the storability potential of Orange Red and Orange Rubis apricots harvested at commercial maturity stage during cold storage (CS) at 1 or 5 °C for up to 28 days, followed by shelf life (SL) at 20 °C for 2 days. The variables evaluated included total soluble solids, titratable acidity, pH only at harvest, weight loss (WL), ethylene production rates, peel color, firmness, chilling injury incidence (CI), concentrations of total phenolics, flavonoids, carotenoids, total antioxidant capacity, b-carotene, b-cryptoxantine, and lutein. The main results showed that storage at 5 °C resulted in higher WL and CI symptoms than at 1 °C during both CS and SL, increased ethylene production during CS, whereas there was limited or no effect of CS temperatures on changes in firmness, color, and all antioxidants during CS. Firmness decreased abruptly soon after harvest in Orange Rubis, but progressively in the remaining samples of both cultivars during CS and SL. SL advanced fruit deterioration according to WL, CI, and softening. During SL, ethylene production increased in all samples. Orange Red exhibited higher ethylene rates during SL and antioxidant concentrations throughout CS and SL, by comparison. Conclusively, storage temperature at 1 °C retained WL, CI, and ethylene production, and both cultivars were marketable up to 21 days CS without SL or up to 14 days CS followed by SL, although Orange Rubis exhibited CI after 14 days, while Orange Red exhibited CI after 21 days of CS.

## 1. Introduction

Apricots (*Prunus armeniaca* L.) are summer fruits that are highly appreciated by consumers due to their unique flavor and aroma combined with their high nutritional value. Apricot cultivation is mainly focused on fresh consumption that produces high income for the growers. However, a considerable part of the produce is consumed as an ingredient in processed products, such as cans, jams, and juice [1].

Apricot cultivars are climacteric fruits [2], and their postharvest life is limited to a few weeks when stored at temperatures near to 0 °C and up to 5 days in shelf-life conditions, depending on cultivar. However, at low temperatures, apricots may exhibit chilling injury (CI) symptoms, such as gel formation in the area around the stone, flesh mealiness, fiber synthesis, internal browning, and flavor loss. CIs can lead to unsalable fruit, and their onset dictates the storage potential of apricots [3,4,5,6]. It is a common practice to harvest apricots at the pre-climacteric stage, even if they have not reached their full flavor potential, when destined for long-term cold storage, transport, and handling [7]. Therefore, optimizing key factors, such as storage temperature and duration, is crucial for long-term fruit storage.

Apricots are an excellent source of carotenoids, which is known for their health promoting properties and are responsible for the fruit’s attractive orange color, making them a delicious and healthy addition in a well-balanced human diet [8]. Carotenoids play an essential role in human vision, b-carotene, a-carotene, and b-cryptoxanthine, which serve as precursors for vitamin A, while lutein and zeaxanthine accumulate in eye macular tissue and possess protective properties against age-related macular degeneration [9,10]. Furthermore, carotenoids and some of their metabolites are proposed to have a protective role in several ROS-mediated disorders, such as heart and neurological diseases and various types of cancers [11]. Apricots are also rich in other antioxidants, such as phenolic compounds and anthocyanins, which are responsible for the red blush in the peel of some cultivars, as well as organic acids, sugars, and minerals [12,13]. It has also been documented that the phenolic compounds of apricots exhibited in vivo cardioprotective properties [14].

In recent years, an important renewal of apricot cultivars is taking place worldwide with the introduction of a large number of new releases in response to productive and industrial changes in the crop. Apricot cultivars with genetic differentiation show different storage-temperature requirements and ripening time. In the present work, we used two commercial apricot cultivars, namely Orange Red and Orange Rubis, as they have specific genetic discriminations: the first one is self-incompatible [15] the second one is considered as self-fertility [16]. Both are medium-early cultivars, with Orange Red producing fruit of good quality suitable both for fresh market and processing, and the apricots having an orange-colored peel with a 20–25% red blush [12,17]. Productivity irregularities have been reported when Orange Red is cultivated under Mediterranean conditions [17]. Orange Rubis is characterized by its oblong fruit shape that is covered with 30–40% red blush; flesh that is aromatic and sweet; and high total soluble solids (TSS), low acidity levels, and medium firmness values [16,18]. The selection of these two cultivars was based on their good quality traits reported in the literature and suggestions by growers due to their qualities, such as fruit weight and color, particularly the red blush and sweet, aromatic flesh.

Since there is a lack of information about the storability potential of these cultivars and in order to investigate the storage temperatures required to avoid chilling-injury development in both apricots cultivars, weight loss (WL), ethylene production rates, peel color, firmness, CI incidence, total antioxidants, namely phenolics (TP), flavonoids (TF), carotenoids (TC) and total antioxidant capacity (TAC) as well as individual carotenes were evaluated at harvest and during refrigerated storage at 1 and 5 °C, at 7-day intervals, for up to four weeks. Also, the changes of the above-mentioned variables were determined in stored fruits after exposure to 20 °C for 48 h. The above storage regimes were chosen as the common industrial storage temperature (1 °C), common domestic storage temperature (5 °C), and shelf-life temperature (20 °C), as well.

## 2. Results

### 2.1. Total Soluble Solids, Titratable Acidity and pH

At harvest, the total soluble solids (TSS) values were 11.6 ± 0.95 and 14 ± 0.53 °Brix, the titratable acidity (TA) values were 0.56 ± 0.10 and 0.52 ± 0.04% malic acid (*w*/*w*), and the pH was 4.10 ± 0.15 and 4.29 ± 0.04 for Orange Red and Orange Rubis apricots, respectively (Table 1).

### 2.2. Weight Loss, Ethylene Production Rates, Fruit Firmness, Peel Color, and CI Incidence

**Figure 1:**
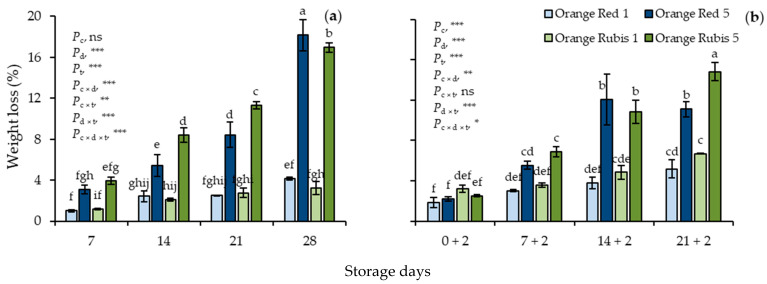
Effects of cultivar and storage temperature on weight loss (**a**) during cold storage (CS) and (**b**) after 2 days shelf life (SL) at 20 °C. Data are means of three replicates ± standard deviations. Columns with different letters are significantly different by Tuckey-HSD test (α = 0.05). Letters correspond to three-way ANOVA of all data either during CS or SL. *P*_c_, probability of cultivar; *P*_d_, probability of storage days; *P*_t_, probability of temperature; *P*_c×d_, *P*_c×t_, *P*_d×t_ and *P*_c×d×t_, probabilities of interactions. *** significant at *p* < 0.001; ** significant at *p* < 0.01; * significant at *p* < 0.05; ns, not significant.

During CS, weight loss (WL) averaged 2.31% on day 7 in all fruit and increased progressively up to day 28. As expected, higher WL values were recorded on apricots stored at 5 °C than on those stored at 1 °C from day 14 to the end of CS (Figure 1a). In detail, WL reached on average 3.7% and 17.6% in fruit stored at 1 and 5 °C, respectively, on day 28. All the effects were highly significant, apart from cultivar. A similar pattern was observed in apricots subjected to 2 days SL (Figure 1b). Particularly, the WL values after 21 days CS and 2 days SL recorded were 5.2% and 11.1% in Orange Red stored at 1 and 5 °C, respectively, and 6.7% and 14.8% in Orange Rubis at 1 and 5 °C, respectively, indicating that the substantial WL difference between the two low-storage temperatures was repeated even after storage and during SL at 20 °C. This difference during SL became considerable after 14 days CS in both cultivars and increased progressively with the CS duration. Also, results showed that fruit of different cultivars behave differently, in detail Orange Rubis apricots stored at 5 °C exhibited higher WL at the end of SL. Indeed, all the effects were significant, except the cultivar × temperature interaction.

**Figure 2:**
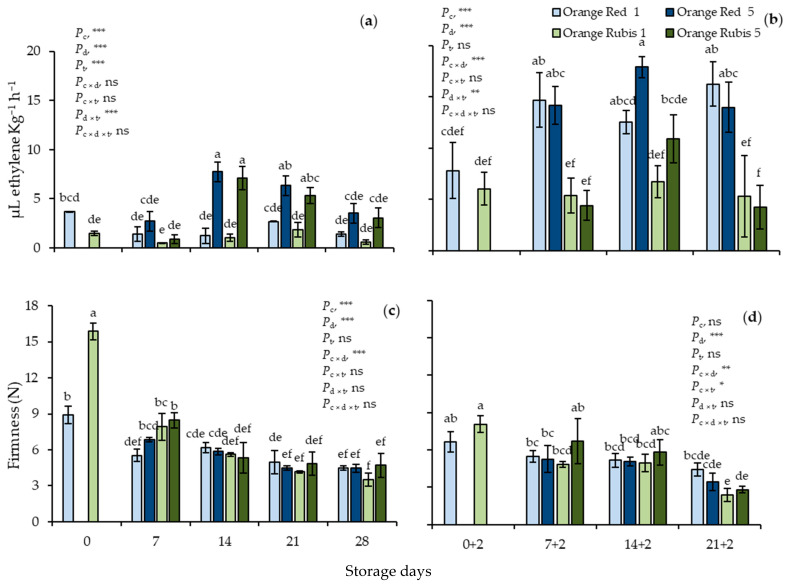
Effects of cultivar and storage temperature on ethylene production rates and firmness, (**a**) and (**c**) during cold storage (CS); (**b**,**d**) after 2 days shelf life (SL) at 20 °C. Data are means of three replicates ± standard deviations. Columns with different letters are significantly different by Tuckey-HSD test (α = 0.05). Letters correspond to three-way ANOVA of all data either during CS or SL. *P*_c_, probability of cultivar; *P*_d_, probability of storage days; *P*_t_, probability of temperature; *P*_c×d_, *P*_c×t_, P_d×t_ and *P*_c×d×t_, probabilities of interactions. *** significant at *p* < 0.001; ** significant at *p* < 0.01; * significant at *p* < 0.05; ns, not significant.

At harvest, C_2_H_4_ production rates were 3.7 and 1.5 μL Kg^−1^ h^−1^ in Orange Red and Orange Rubis fruit, respectively (Figure 2a). Thereafter, C_2_H_4_ values in CS at 5 °C in fruit of both cultivars increased significantly on day 14, reaching 7.4 μL Kg^−1^ h^−1^ on average, remained at similar levels on day 21, and then decreased to 3.25 μL Kg^−1^ h^−1^. In contrast, at 1 °C all values of C_2_H_4_ production were similar to those at harvest. All the effects were highly significant, apart from cultivar × storage days, cultivar *×* temperature, and cultivar *×* storage days × temperature interactions. Partial analysis of both cultivars on days 0 and 0 + 2 showed that exposure to 20 °C resulted in significant increase in both cultivars, and the C_2_H_4_ production rates reached 7.8 and 6 μL Kg^−1^ h^−1^ in Orange Red and Orange Rubis apricots, respectively (Figure 2b). Thereafter, in all Orange Red samples, ethylene values elevated sharply on day 7 + 2, reaching 16.4 μL Kg^−1^ h^−1^ on average, and remained at almost stable levels throughout storage. On the other hand, in all Orange Rubis fruit and on all sampling days of SL, C_2_H_4_ production rates were 6.14 μL Kg^−1^ h^−1^ on average. The effect of cultivar and storage days, along with the interactions of cultivar × storage days and storage days × temperate were estimated to be significant.

The initial firmness values were 8.9 and 15.9 N for Orange Red and Orange Rubis apricots, respectively, decreasing rapidly during the first week of CS and then remaining at almost stable levels until the end of the CS, reaching 4.3 N on average in fruit of both cultivars (Figure 2c). Similar firmness values were observed in all apricots from day 14 and afterwards during CS regardless of storage temperature or cultivar. The only significant effects were those of cultivar, storage days, and their interaction. During SL, a similar pattern of firmness changes to those during CS were observed on fruit, and after 21 days CS and 2 days SL, firmness values averaged 3.3 N for all samples. The effect of storage days, as well as the interactions of cultivar × storage days and cultivar × temperature were evaluated to be significant. Partial analysis of the initial data from both CS and SL confirmed the significant effect of cultivar, exposure to 20 °C, and their interaction. Firmness in Orange Rubis was significantly higher at harvest, but decreased dramatically thereafter, reaching levels similar to Orange Red.

At harvest, the color parameter L* was 52.25 and 44.79 for Orange Red and Orange Rubis fruit, respectively, and no significant changes were observed during CS (Table 2). The effects of cultivar, storage days, and their interaction were significant. Initial differences in L* values between the two cultivars faded away after 2 days SL at the beginning of storage (Table 3), and any changes thereafter were negligible. As a result, no factor had a significant effect on the L* color parameter.

The initial values of hue angle averaged 51.8 in fruit of both cultivars and remained almost stable during CS (Table 2), and the significant effects observed were only from days and from the cultivar x days interaction. After 2 days of SL at the beginning of the experiment, Orange Red apricots exhibited values of *hue angle* ~50, while those of Orange Rubis were ~57 (Table 3). During SL, factorial analysis showed the significant effect of cultivar, storage days, and their interaction.

In Orange Red apricots, the parameter C* was initially evaluated 44.8, but no significant changes were observed thereafter, with C* values averaging 42.5 for all sampling days in CS (Table 2). In Orange Rubis, values were also similar throughout CS, averaging 34. The differences observed in the color parameter C* between the two cultivars were significant on each sampling date during CS and the effect of cultivar and the interaction of cultivar × storage days were also significant. A similar pattern of differences in the parameter C* between the fruit of the examined cultivars appeared in apricots subjected to SL conditions in all sampling dates (Table 3). The effects of cultivar and storage days were estimated to be significant. Partial analysis of color parameters on days 0 and 0 + 2 showed no effect of cultivar or exposure to 20 °C for 2 days for both L* and hue, but these factors were highly significant on C* in both cultivars.

CIs occurred after 21 and 14 days of CS in Orange Red and Orange Ruby apricots, respectively (Table 4). The highest percentage of fruit with CI symptoms observed in Orange Red apricots stored at 5 °C for up to 28 days. Factorial analysis revealed the significant effect of storage days and temperature, and of the interactions of cultivar × storage days and storage days × temperature. As expected, the percentage of the apricots with CIs was higher after SL than CS. In detail, CI incidence was estimated to be ~1.9% and 12% after 14 days CS followed by 2 days SL in Orange Red and Orange Rubis fruit, respectively, while after 21 days CS and 2 days SL the percentage of apricots with CIs reached 23.6% and 11.3% in Orange Red and Orange Rubis, respectively. The effects of storage days and the interaction of cultivar × storage days were significant. However, it has to be noted that CI recordings were at limited areas and of low intensity up to 21 days during CS or 14 days CS and 2 days SL (14 + 2).

### 2.3. Total Phenolics (TP), Total Flavonoids (TF) and Total Antioxidant Capacity (TAC)

**Figure 3:**
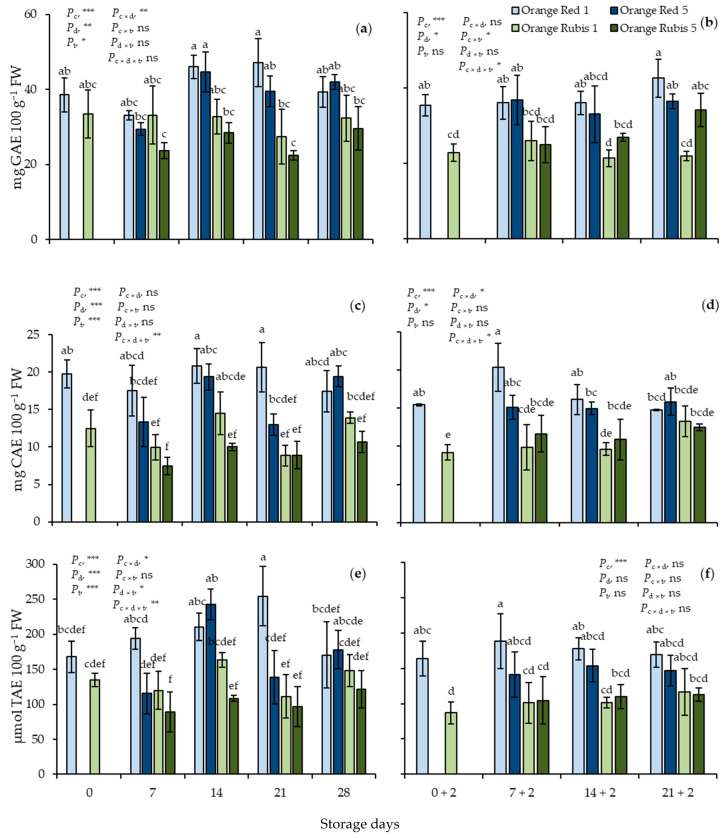
Effects of cultivar and storage temperature on total phenolics (**a**,**b**), total flavonoids (**c**,**d**), and total antioxidant capacity estimated by FRAP assay (**e**,**f**), (**a**,**c**,**e**) during cold storage (CS) and (**b**,**d**,**f**) after 2 days shelf life (SL) at 20 °C. Data are means of three replicates ± standard deviations. Columns with different letters either during CS or SL are significantly different by Tuckey-HSD test (α = 0.05). Letters correspond to three-way ANOVA of all data. *P*_c_, probability of cultivar; *P*_d_, probability of storage days; *P*_t_, probability of temperature; *P*_c×d_, *P*_c×t_, *P*_d×t_ and *P*_c×d×t_, probabilities of interactions. *** significant at *p* < 0.001; ** significant at *p* < 0.01; * significant at *p* < 0.05; ns, not significant.

At harvest, in Orange Red and Orange Rubis fruit, the TP values were 38.6 and 33.4 mg GAE 100 g^−1^ FW, respectively; TF, 19.7 and 12.5 mg CAE 100 g^−1^ FW, respectively; and TAC, 167.7 and 134.9 μmol TAE 100 g^−1^ FW, respectively (Figure 3a,c,e). During CS, few, but significant, differences in the TP values were observed in the two cultivars, although the effects of cultivar, storage days, and temperature, along with the interaction of cultivar × storage days were significant. Indeed, the analysis showed that TP values in Orange Red were higher than in Orange Rubis at 1 °C higher than at 5 °C, while values were low on day 7. During CS, the patterns of changes in TF and TAC values were similar to those of TP. In detail, the concentration of TF was estimated at higher levels in Orange Red apricots than in Orange Rubis initially, and no significant changes occurred in TF in each cultivar in comparison to their initial values. Moreover, the values of TF were significantly influenced by cultivar, storage days, temperature, and the interaction of all three factors. Concerning TAC, the initial values were not significantly different between the cultivars but became so afterwards with the increases in Orange Red up to day 21, while few changes occurred in Orange Rubis during storage. All factors, apart from the interaction of cultivar × temperature, significantly influenced the values of TAC, with the averaged values in Orange Red being higher than those in Orange Rubis and storage at 1 °C resulting in higher TAC than at 5 °C on average.

During SL, the differences observed in the values of the three above-mentioned variables were retained. Orange Red exhibited significantly higher values of TP, TF, and TAC than Orange Rubis (Figure 3b,d,f). Particularly, in Orange Red apricots, concentrations of TP, TF, and TAC were found at 36.8 mg GAE 100 g^−1^ FW, 16.1 mg CAE 100 g^−1^ FW, and 162.9 μmol TAE 100 g^−1^ FW, respectively, on average for all sampling dates of SL. On the other hand, in Orange Rubis fruit, TP, TF, and TAC values averaged 22.8 mg GAE 100 g^−1^ FW, 11 mg CAE 100 g^−1^ FW, and 104.7 μmol TAE 100 g^−1^ FW, respectively, in all sampling dates of SL. Any changes observed in concentrations of the three determined traits in each cultivar during SL were not significant. Factorial analysis showed the highly significant effect of cultivar on all three determined variables. Also, TP values were influenced significantly by storage days, the interactions of cultivar × temperature and cultivar × storage days × temperature, the TF values by storage days, and the interactions of cultivar × storage days and cultivar × storage days × temperature, whereas cultivar was the only significant factor for TAC.

Partial analyses of the initial values of both cultivars on days 0 and 0 + 2 confirmed the higher TP, TF, and TAC levels of Orange Red and the decreases in TP and TF of Orange Red, as well as in TAC of Orange Rubis.

### 2.4. Total Carotenes (TC) and Individual Carotenoid Compounds

**Figure 4:**
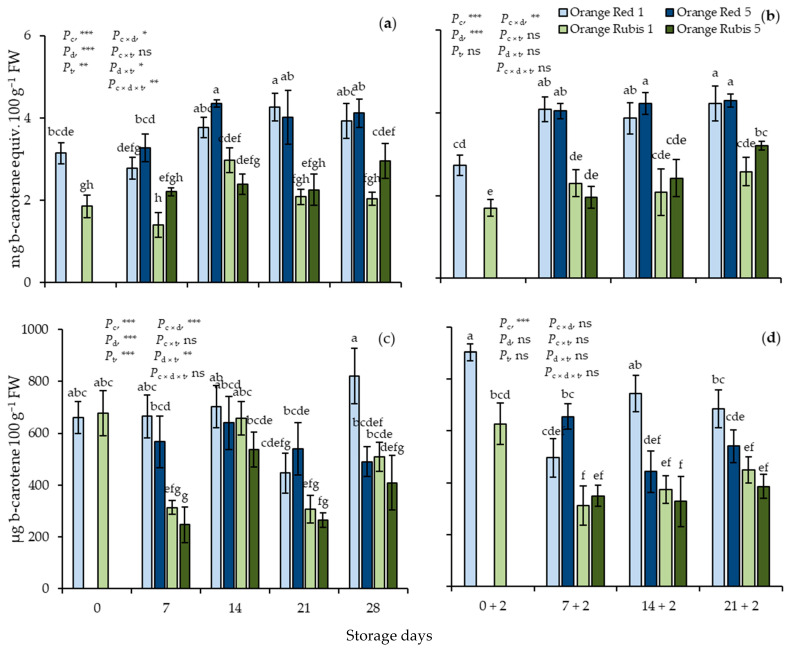
Effects of cultivar and storage temperature on total carotenoids (**a**,**b**) and b-carotene (**c**,**d**) (**a**,**c**) during cold storage and (**b**,**d**) after 2 days shelf life at 20 °C. Data are means of three replicates ± standard deviations. Columns with different letters, either during CS or SL, are significantly different by Tuckey-HSD test (α = 0.05). Letters correspond to three-way ANOVA of all data. *P*_c_, probability of cultivar; *P*_d_, probability of storage days; *P*_t_, probability of temperature; *P*_c×d_, *P*_c×t_, *P*_d×t_ and *P*_c×d×t_, probabilities of interactions. *** significant at *p* < 0.001; ** significant at *p* < 0.01; * significant at *p* < 0.05; ns, not significant.

At harvest, TC concentration was significantly higher in Orange Red fruit compared to Orange Rubis and estimated at 3.1 and 1.9 mg b-carotene equiv. 100 g^−1^ FW, respectively (Figure 4a). During CS the differences in TC concentrations between the fruit of the two cultivars were maintained, mainly due to the increased values in Orange Red from day 14 to the end of storage at both low temperatures, whereas in Orange Rubis the values either remained fairly stable or exhibited limited increases. All the effects were significant apart from the interaction of cultivar × temperature. Also, on each sampling date of SL TC levels in Orange Red fruit were significantly higher than the respective levels in Orange Rubis (Figure 4b). The effects of cultivar and storage days, along with their interaction were significant. The transfer of harvested fruits at 20 °C for 2 days resulted in small, but significant, decreases, whereas the difference between the cultivars were retained, as confirmed by partial analysis.

As expected, b-carotene was the major carotenoid compound compromising 98% of the total carotenoids quantified using HPLC in fruit of both cultivars. The initial levels of b-carotene were similar in apricots of both cultivars and averaged ~670 μg 100 g^−1^ FW (Figure 4c). However, on day 7, Orange Rubis exhibited decreased values of b-carotene at both low temperatures, and the differences between the cultivars became significant. All the effects were significant apart from the interactions of cultivar × storage temperature and cultivar × storage days × storage temperature. During SL, values in Orange Red increased significantly and substantially during two days at 20 °C immediately after harvest, reaching 903 μg 100 g^−1^ FW, as also confirmed by partial analysis, while they showed decreases thereafter, independent from the previous CS temperature. Orange Rubis, on day 0 + 2 exhibited values similar to harvest, but the values declined in the fruit regardless of CS temperature, remaining consistently lower than those of Orange Red (Figure 4d). The effect of cultivar was the only significant factor for these changes.

b-cryptoxanthine was evaluated to be 6.3 μg 100 g^−1^ FW, on average, in apricots of both cultivars during CS and SL (Table 2 and Table 3), and any changes observed during CS or SL were negligible. b-cryptoxanthine values were influenced significantly by cultivar during CS and SL, and by storage days during CS. Lutein averaged 8 and 4.7 μg 100 g^−1^ FW in Orange Red and Orange Rubis, respectively, during CS (Table 2), and the effect of cultivar was the only significant one. Some fluctuations in lutein concentration during SL were small (Table 3), and the effect of storage days was the only significant factor.

## 3. Discussion

### 3.1. Total Soluble Solids, Titratable Acidity and pH

TSS and TA are considered key quality indices due to their direct effect on the fruit’s overall taste. Here, Orange Rubis fruit exhibit higher TSS and pH, and lower TA values compared to those mentioned in the literature for apricots of the same cultivar [16,18,19], indicating that fruit were harvested at a more advanced mature stage than fruit in the above-mentioned studies. In addition, Piagiani et al. [18] mentioned that Orange Rubis apricots were highly rated by a group of 18 trained panelists. Drogoudi et al. [12] reported higher TSS and TA and lower pH values in Orange Red apricots than our study. Although there are no particular TSS, pH, and TA values in legislation standards for apricot harvest and marketing, fruit of acceptable quality for consumers should have a minimum TSS value of 10% and a TA value not exceeding 1% (*w*/*w*) [20,21]. In our study, apricots of both cultivars complied with these requirements. Furthermore, Guillén et al. [22] and Ruiz and Egea [23] observed a significant variation in TSS values between two consecutive years.

### 3.2. Weight Loss, Peel Color, Fruit Firmness, Ethylene Production Rates, and CI Incidence

The weight of the apricot fruit is one of the critical quality traits that directly influences farmers’ income. At harvest, the weight of fruit averaged 60 g and 78 g for Orange Red and Orange Rubis, respectively, indicating apricots of large weight and size. The present results are in line with other studies on ‘Orange Red’ [12] and ‘Orange Rubis’ [19]. Moreover, WL is considered one of the main deteriorating factors in apricots during storage. As expected, WL during CS was higher at 5 than at 1 °C, and a similar pattern was observed during SL. However, in both cultivars, it was of interest that fruit kept in ‘memory’ the temperature of their previous CS and continued to exhibit higher WL at 20 °C after CS at 5 than at 1 °C. This could be attributed to integrity loss of epidermal and flesh cells, facilitating the water loss that, in turn, affected other determined variables, such as firmness and disorders in fruits stored at 5 °C at the end of SL. For two consecutive years, Ezzat et al. [24] reported WL values ranging 2.5 to 8.2% in apricots of ten cultivars stored at 3 °C for up to 28 days, but this study does not report on packaging conditions during storage. Another detailed study concerning apricot weight loss [25] showed that the percentage and distribution of perforation in the packaging (clamshells) play an important role in water loss and, consequently, WL. Overall, according to present results, on day 28, immediately after removal from store and after 21 days in CS and 2 days SL (21 + 2), all apricots became nonmarketable.

At harvest, Orange Red apricots had considerably higher L* and C* values compared to Orange Rubis fruit, while *h°* values were measured at similar levels in fruit of both cultivars. According to Ruiz et al. [26] the above-mentioned values in color parameters correspond to apricots with orange peel and flesh that have obtained a red blush. The values of color parameters measured at the current study are in good accordance with other studies in Orange Red [12] and Orange Rubis fruit [16].

Fruit firmness is also considered as an important quality attribute of the apricot fruit and it highly related to their potential of long-term storage, distribution to far markets and shelf life at ambient temperatures. Fruit firmness, along with peel color and TSS are key quality indices that affect consumers’ acceptance [27] and determine when fruits have reached commercial maturity [12,20]. Apricots are suitable for harvest when their flesh is still firm and the color of the peel changes from green to yellow-orange [5]. According to the above-mentioned criteria and considering TSS and TA values, apricots of both cultivars were harvested at the recommended commercial stage. Here, apricots at harvest exhibited firmness values of ~9 and 16 N for Orange Red and Orange Rubis, respectively. However, a sharp decrease in firmness values by 44% on average in fruit of both cultivars was observed during the first 7 days of CS. A similar decrease during the first days of storage was reported in Farbaly apricots stored at 1 °C, as reported in our previous study [1]. Comparable significant decreases in firmness values have been described by Egea et al. [28] in Búlida apricots stored at 2 °C for 21 days and then subjected to 4 days at 20 °C. In addition, Gullién et al. [22] observed a similar to our study firmness loss, indicating that apricot shelf life is limited to 3 days.

At harvest, apricots of both cultivars exhibited rather low and similar rates of ethylene, indicating that fruit were harvested at or close to the beginning of the climacteric stage. During CS at 1 °C, ethylene production was evaluated at rather stable levels; however, increases in ethylene rates were observed in apricots stored at 5 °C on day 14 and afterwards. Several studies have reported relatively low and stable ethylene rates in apricots stored at 1 and 2 °C, indicating that low storage temperatures are good means of delaying the ripening processes stimulated by ethylene [29,30]. Nevertheless, there are cultivars for which ethylene does not promote firmness decreases, although the ethylene action inhibitor 1-MCP prevented these losses [31]. As expected here, the ethylene production rates increased in apricots subjected to 2 days at SL after harvest, regardless of cultivar. Moreover, in Orange Red fruit, ethylene levels increased further during SL and after a week of CS and remained at stable levels until the end of SL. In contrast, Orange Rubis exhibited lower ethylene levels at CS and smaller increases during SL than Orange Red. These findings are in general accordance with those of other studies concerning apricot postharvest behavior during cold storage and shelf life [22,28,29,32]. Alvarez-Hernadez et al. [29] reported that apricots stored at 15 °C reached the climacteric peak on day 14 of storage, coinciding with the highest production level, contrary to fruit stored at 2 °C, which exhibited low and stable levels. Moreover, it has been observed that cultivar along with fruit maturity stage at harvest have a significant effect on ethylene production levels during storage [30]. In the literature, an ethylene-free environment is recommended during the storage and transportation of apricots to reduce softening enhanced by ethylene [33]. New technologies could be beneficial to extend the storage duration and improve the quality of stored apricots [34,35].

It is well established that stone fruit, including apricots, may exhibit chilling injuries and internal breakdown when stored at a temperature range of 0–10 °C, which limits their postharvest life to a few weeks. On the contrary, the use of higher temperatures between 15 and 27 °C allow for fruit ripening [36]. Chilling injuries, such as gel formation in the area around the stone, flesh mealiness, and internal browning render fruit unsalable and determine the storability of apricots of different cultivars [5,37]. Here, CIs first occurred after 14 days of CS in Orange Rubis apricots, regardless of storage temperature. However, the percentage of CI was higher when apricots subjected to SL after 21 days of CS. Manolopoulou and Mallidis [38] suggested that storage temperatures between 5 and 7 °C lead to mealy fruit that lack flavor and proposed the use of temperatures around 0 °C. On the other hand, Dong et al. [3] reported flesh mealiness in fruit stored in temperatures near 0 °C. Consequently, the choice of the suitable storage temperature for each cultivar is essential and has a direct effect on the storage potential of fresh apricots.

### 3.3. Total Antioxidants and Total Antioxidant Capacity

Total antioxidants concentration along with total antioxidant capacity assessment are key factors that relate the consumption of apricots to health-promoting benefits. The present results at harvest comply well with other studies on ‘Orange Rubis’ [16] and are within the range proposed by Kafkaletou et al. [20] in apricots of eight cultivars grown in Greece. In a large-scale study, the levels of TP ranged between 62 and 80 mg 100^−1^ FW in the edible portion of 37 apricot cultivars [13]. In the literature, there is a wide range in values of TP, TF, and TAC, and a significant effect of genotype and harvest season has been ascribed to these values [19,27,39].

In the present study, it is of interest that similar patterns of changes were observed in TP, TF, and TAC during CS and SL. In detail, no significant differences were evaluated in the values of TP and TAC in each cultivar during CS, and any fluctuations occurred were limited. Likewise with this work, Cambell et al. [40], Kafkaletou et al. [1], and Leccesse et al. [27,39] suggested that apricots stored at low temperatures maintained constant values of TP and TAC. However, Orange Rubis apricots exhibited significantly lower values in the determined variables of TP, TF, and TAC compared to Orange Red fruit when subjected to SL right after harvest. In another study on NS-4 apricots stored at 1 °C for 21 days, the levels of TP and TF were found at almost stable levels but decreased after SL at 24 °C for 3 days [41]. Contrarily, Ezzat et al. [42] reported that the levels of TP and TAC, evaluated using FRAP assay, decreased during CS at 1 °C for up to 21 days and during SL at 25 °C for up to 8 days. However, it is noteworthy that the concentrations expressed per fresh weight, as here, are valuable from the perspective of the retention of nutritional value of fruit, whereas expression per dry weight are related to net synthesis or degradation of the determined compounds.

### 3.4. Total Carotenes and Individual Carotenoid Compounds

At harvest, Orange Red apricots exhibited higher values of TC than Orange Rubis ones, and this difference was maintained during CS and SL. Moreover, an increase in TC levels was measured in Orange Red fruit during SL, this increase can be attributed, to some extent, to WL. In another study in Bergarouge apricots, TC concentration increased during CS at 1 °C for 14 days and then decreased at day 21, while when fruit were subjected to SL, TC values remained at constant levels until day 4 and then decreased on day 8 [42]. Also, Milović et al. [41] reported an increase in TC concentration in NS-4 apricots during CS at 1 °C for 21 days and after 3 days of SL. Ruiz et al. [26] reported that TC values ranged between 1.3 and 38.5 mg 100 g^−1^ FW and observed a significant relationship between flesh color and TC concentration in 37 apricot cultivars, proposing that the use of color parameter values, particularly *h°*, can lead to the prediction of TC content of apricot cultivars.

The chromatographic analysis of carotenes revealed that b-carotene was the dominant carotenoid compound, comprising 98% of the compounds quantified, followed by b-cryptoxanthine and lutein that both evaluated at similarly low levels. It is well established that b-carotene is the major carotenoid compound in apricots, and the present results are in line with other studies [20,26,40,43]. At harvest, the concentration of b-carotene was estimated to be at similar levels in fruit of both cultivars examined. However, the values of b-carotene decreased in Orange Rubis apricots after 7 days of CS and remained at stable levels thereafter; on the contrary, b-carotene concentration was measured at similar levels throughout CS in Orange Red fruit apart from a decrease observed in apricots stored at 5 °C for 28 days, but on that day the fruit were inedible. The changes observed in the levels of b-cryptoxanthine and lutein during CS and SL did not follow a particular pattern and any changes observed were negligible. Cambell et al. [40] reported that b-carotene and b-cryptoxanthine concentration increased by 5- and 2-fold, respectively, after 4 weeks of CS at 1 °C in Hargrand apricots, while lutein maintained at constant levels.

## 4. Materials and Methods

### 4.1. Fruit Material and Storage

The apricot (*Prunus armeniaca* L.) fruit of two cultivars ‘Orange Red’ and ‘Orange Rubis^®^’ were hand-picked from trees cultivated in a commercial orchard located in Argolida, Greece. Apricots, at an advanced commercial stage of maturity matching consumers’ preference and according to the recommendations and practices of local orchards, were harvested during early morning on 11 June 2019 and transported to the laboratory within 3 h. Once in the laboratory, fruit free from visual defects and uniform in peel color and shape were placed in commercial vented clamshells (18 cm × 14 cm × 6 cm). Each clamshell contained 6 and 5 apricots from ‘Orange Red’ and ‘Orange Rubis’, respectively, and served as a replicate. Half of the clamshells were stored in a cold chamber at 1 °C and 90% RH, while the other half stored at 5 °C and 90% RH. Quality and biochemical traits were determined before storage at day 0, and after 7, 14, 21 and 28 days of cold storage (CS) either at 1 or 5 °C, as well as after the stored fruits were exposed to 20 °C for two days (shelf life). Determinations were carried out on 3 replicates per cultivar and storage temperature on each sampling date. Apricots from the last sampling date (28 d) were not subjected to shelf life. All evaluations after CS were performed after fruit temperature equilibration at 20 °C for 15 h, apart from weight loss, which was measured immediately after apricots were removed from storage in the clamshell. At each sampling date, soon after the quality index measurements, slices of all apricots of each replicate were dipped in liquid nitrogen and then stored at −20 °C until the extraction of phytochemicals within one month.

### 4.2. Total Soluble Solids (TSS), Titratable Acidity (TA), and pH

TSS, TA, and pH were evaluated in the fruit’s juice. In detail, 2 slices per apricot were homogenized in a food processor and centrifuged at 4000× *g* for 5 min to obtain a clear juice. TSS values were recorded by a digital refractometer (HI 96801, Hanna Instruments Inc., Woonsocket, RI, USA) using temperature compensation mode and expressed as °Brix. TA was measured by titration with 0.1 N NaOH to pH 8.2 and expressed as malic acid equivalents (%) *w*/*w*. All assays were performed twice per replicate.

### 4.3. Weight Loss (WL), Ethylene Production Rates, Fruit Firmness, Peel Color, and Chilling Injury (CI) Incidence

Fruit WL was monitored during storage. Fruit weight was recorded on each sampling date, and the percentage of changes was calculated in respect to values of day 0 (%, *w*/*w*) at each temperature and shelf life.

Ethylene production rates were estimated using gas chromatography according to Kafkaletou et al. [1] with some modifications. Apricots were incubated in 1.5 L jars at 20 °C for up to 2 h, and a headspace sample of 1 mL was analyzed by a Perkin-Elmer-Sigma 300 gas (Perkin-Elmer, Norwalk, CA, USA) chromatograph equipped with a flame ionization detector and an alumina activated column (120 × 0.2 cm i.d. 20–100 mesh) (Restek, Bellefonte, PA, USA). Ethylene rates were expressed as μL Kg^−1^ h^−1^.

Firmness was evaluated using an HD-Plus texture analyzer (Stable Micro Pedicels Ltd., Godalming, UK) and Texture Expert Exceed Software for the data analysis [44]. The evaluation of the textural traits of whole apricots was performed with a stainless plate of 25 × 60 mm, the compression depth was set at 5 mm, movement speeds at 1 mm s^−1^ during the test, 5 mm s^−1^ for the pre-test and 10 mm s^−1^ for the post-test and. The measurement was conducted at the equatorial zone in each fruit, and the results were expressed as the maximum recorded force in Newtons (N).

Peel color was recorded by using a Minolta chroma meter (CR-300; Minolta, Ahrensburg, Germany) on two opposite points on the equatorial zone of each apricot per replicate. Results are expressed as lightness (L*, ranging from 0 = black to 100 = white), hue angle (*h°*), and chroma (C*).

Each apricot, after being cut in halves, was visually examined for CI incidence visually. CI symptoms were depicted by the presence of brown areas in fruit flesh [45], and the absence (0) or presence (1) of symptoms was expressed as the mean of fruits per replicate (6 or 5 apricots for ‘Orange Red’ or ‘Orange Rubis’, respectively).

### 4.4. Total Phenolics (TP), Total Flavonoids (TF) and Total Antioxidant Capacity (TAC)

Antioxidants extractions and measurements were carried out according to Kafkaletou et al. [20]. Briefly, frozen slices of each replicate were homogenized with 80% acetone in DDW (2.5 mL g^−1^ tissue) in a laboratory blender. The homogenate was incubated in a supersonic bath at 4 °C for 15 min under darkness, then centrifuged at 4000× *g* for 5 min. This process was repeated thrice. The recovered supernatants were used for TP, TF, and TAC assays. TP were determined via Folin–Ciocalteu method using a spectrophotometer (Heλios Gamma and Delta, Spectronic Unicam, Cambridge, UK) to record the absorbance at 750 nm versus a blank. TF values were assessed using the colorimetric method proposed by Gunes et al. [46], and TAC were assessed by ferric-reducing antioxidant power (FRAP) [47]. For all evaluations, duplicate reactions per replicate were implemented, and the results of TP, TF, and TAC were expressed as equivalents of gallic acid (GAE), catechin (CAE), and trolox acid (TAE), respectively, all on a fresh weight basis.

### 4.5. Total Carotenes (TC) and Individual Carotenoid Compounds

TC were extracted and measured following the procedure proposed by Kafkaletou et al. [20]. Specifically, the extraction was conducted by homogenizing frozen apricot slices with a solution acetone/hexane (4/6 *v*/*v*) (6 mL g^−1^ tissue) using an Ultra-Turrax T25 (IKA Labortechnik, Staufen, Germany) at 9500 rpm for 1 min. The homogenate was placed in an orbital shaker set at 450 rpm for 30 min under darkness, then centrifuged at 4000× *g* for 5 min. The procedure was repeated four times. The absorbance of the combined supernatants was read at 453, 505, 663, and 645 nm, and the results were expressed as b-carotene equivalents on a fresh weight basis [48].

Individual carotenoid compounds were extracted and determined using the method of Ruiz et al. [13] with some modifications [20]. In detail, frozen tissue was added to a solution of methanol/hexane (1/1 *v*/*v*) (2 mL g^−1^ tissue) and homogenized in ice using an Ultra-Turrax at 9500 rpm for 2 min and then centrifuged at 4000× *g* at 4 °C for 5 min to recover supernatant. The above-mentioned process was repeated three more times using 5 mL of hexane instead of the methanol/hexane solution. Carotenoids were extracted three times from the combined supernatants using an equal volume of ethyl acetate; the organic solvent was evaporated under N_2_ flow at 37 °C; and the residue was dissolved in 1 mL acetone (HPLC grade), passed through a nylon syringe filter (0.2 μm pore size), and analyzed using an HPLC system within 12 h. The HPLC system consisted of a pump Nexera X2 (LC-30 AD), an autosampler (SIL-30 AC), and a diode-array detector (SPD-M20A) (Shimadzu, Kyoto, Japan). The separation was performed by a Pursuit XRs RP-18 analytical column (250 × 4 mm; 5 μm particle size) under 1.5 mL min^−1^ flow rate at 37 °C, and the injection volume was 20 μL. The elution solvents were acetone (A) and water (B), and the gradient program started with 85% A, then 100% A at 15 min, 85% A at 16 min, and remained at 85% A for 7 min. The carotenoid compounds were identified by comparison of retention times and UV–Vis spectra of samples with those of authentic standards (b-carotene, b-cryptoxanthine, and lutein) (Extrasynthese, Genay, France), quantification was achieved by constructing a multi-point calibration curve for the corresponding standard, and the results are expressed as μg 100 g^−1^ on a fresh weight basis. The data analyses were performed using LabSolutions LC/GC 5.82 software (SkyCom, Kyoto, Japan).

### 4.6. Data Analysis

The results were analyzed during storage and separately during shelf life by three-way ANOVA, with the three factors being the cultivar, storage days, and storage temperature. When denoted, two-way ANOVA analyses were also carried out between 0 and 0 + 2 SL of the two cultivars. Mean (of three replicates of 6 and 5 apricots each of ‘Orange Red’ and ‘Orange Rubis’, respectively) comparisons were performed using the Tuckey-HSD test (α = 0.05). Standard deviation values were calculated from the residual variances. All statistical analyses were conducted using JMP 7.0.1 software (SAS Institute, Cary, NC, USA).

## 5. Conclusions

The results here show that at harvest Orange Red exhibited higher values of all three color parameters, TF, and TC, while Orange Rubis had higher TSS and firmness values, by comparison. Moreover, apricots of both cultivars stored at 1 °C showed lower WL and CI symptoms than at 5 °C during both CS and SL, and stable and low ethylene production rates during CS, whereas storage temperature did not influence the changes in firmness, color, and any antioxidants during CS. A sharp decrease in firmness was observed soon after harvest in Orange Rubis, but in the remaining samples of both cultivars gradual decreases were measured during CS and SL. The fruit deterioration according to WL, CI and softening was promoted by SL. Also, ethylene production rates increased in all samples during SL, and Orange Red had higher ethylene rates during SL and antioxidants concentrations throughout CS and SL by comparison. In conclusion, fruit of both cultivars stored at 1 °C were evaluated with lower values of WL, CI, and ethylene production rates compared with those stored at 5 °C. Apricots of both cultivars were marketable up to 21 days CS without SL or up to 14 days CS followed by SL, although Orange Rubis showed CI after 14 days, while Orange Red showed CI after 21 days of CS. Also, it is of great importance that there was no reduction in TC either at 1 °C or at 5 °C, as carotenoid compounds are highly sensitive to environmental conditions. Finally, it seems likely that genotype greatly influences morphological and organoleptic characteristics of apricot cultivars rather than storage regimes.

## Figures and Tables

**Table 1:** Total soluble solids (TSS), titratable acidity (TA), and pH in apricots at harvest.

Cultivar	TSS (°Brix)	TA (% Malic Acid, *w*/*w*)	pH
Orange Red	11.60 ± 0.95 ^1^	0.56 ± 0.10	4.10 ± 0.15
Orange Rubis	14.00 ± 0.53	0.52 ± 0.04	4.29 ± 0.04

^1^ Mean values of three replicates ± standard deviations.

**Table 2:** Effects of cultivar and storage temperature on peel color parameters (L*, *hue angle*, and C*) and individual carotenoid compounds (b-cryptoxanthine and lutein) during cold storage (CS).

			Storage Days					Probabilities						
	Cultivar	Temperature (°C)	0	7	14	21	28	*P*_c_	*P*_d_	*P*_t_	*P*_c×d_	*P*_c×t_	*P*_d×t_	*P*_c×d×t_
Peel color parameters														
L*	Orange Red	1	52.25 ± 1.53 ab ^1^	44.55 ± 3.16 b	48.69 ± 4.14 ab	46.01 ± 2.81 ab	54.12 ± 1.02 a	***	*	ns	**	ns	ns	ns
		5	52.25 ± 1.53 ab	52.72 ± 2.61 ab	50.05 ± 4.63 ab	45.42 ± 3.66 ab	52.58 ± 4.50 ab							
	Orange Rubis	1	44.79 ± 2.19 b	46.03 ± 0.88 ab	48.57 ± 1.85 ab	47.84 ± 2.74 ab	46.11 ± 0.65 ab							
		5	44.79 ± 2.19 b	48.60 ± 0.75 ab	49.39 ± 5.74 ab	46.54 ± 1.35 ab	48.43 ± 3.30 ab							
*h°*	Orange Red	1	59.87 ± 1.89	44.06 ± 6.78	51.94 ± 9.35	46.96 ± 3.86	62.00 ± 2.95	ns	*	ns	*	ns	ns	ns
		5	59.87 ± 1.89	58.63 ± 6.07	53.34 ± 8.19	48.44 ± 6.36	59.75 ± 9.17							
	Orange Rubis	1	51.76 ± 3.92	53.77 ± 2.19	60.18 ± 4.61	57.06 ± 6.78	55.02 ± 1.54							
		5	51.76 ± 3.92	60.36 ± 2.22	59.96 ± 11.60	51.12 ± 5.41	59.86 ± 12.51							
C*	Orange Red	1	44.81 ± 0.48 ab	41.43 ± 1.47 abc	41.67 ± 1.90 abc	41.41 ± 2.54 abc	45.27 ± 0.55 a	***	ns	ns	**	ns	ns	ns
		5	44.81 ± 0.48 ab	44.07 ± 1.67 ab	42.76 ± 3.06 ab	38.47 ± 2.76 bcd	43.85 ± 3.51 ab							
	Orange Rubis	1	34.21 ± 0.91 de	32.87 ± 2.50 de	34.36 ± 0.41 de	35.54 ± 1.97 cde	31.52 ± 3.59 e							
		5	34.21 ± 0.91 de	33.63 ± 1.16 de	34.92 ± 4.78 cde	35.09 ± 0.83 cde	33.53 ± 0.28 de							
Carotenes														
b-cryptoxanthine (μg 100 g^−1^ FW)	Orange Red	1	6.50 ± 3.09 ab	6.16 ± 1.29 ab	10.10 ± 2.76 a	5.10 ± 1.22 ab	7.72 ± 0.63 ab	***	*	ns	ns	ns	ns	ns
		5	6.50 ± 3.09 ab	6.44 ± 1.51 ab	6.14 ± 2.50 ab	8.96 ± 3.63 ab	6.44 ± 1.48 ab							
	Orange Rubis	1	5.30 ± 0.97 ab	3.15 ± 1.03 b	7.68 ± 2.01 ab	3.97 ± 1.32 ab	3.83 ± 3.65 ab							
		5	5.30 ± 0.97 ab	2.50 ± 1.34 b	5.36 ± 3.77 ab	2.82 ± 1.04 b	3.83 ± 0.69 ab							
Lutein (μg 100 g^−1^ FW)	Orange Red	1	8.66 ± 3.57	9.17 ± 5.57	7.94 ± 2.74	7.07 ± 3.15	7.57 ± 4.43	***	ns	ns	ns	ns	ns	ns
		5	8.66 ± 3.57	9.25 ± 2.38	3.32 ± 1.74	10.92 ± 6.38	7.62 ± 5.96							
	Orange Rubis	1	7.28 ± 2.48	3.98 ± 0.47	3.84 ± 1.77	6.93 ± 1.71	3.38 ± 1.34							
		5	7.28 ± 2.48	3.50 ± 2.20	3.35 ± 1.05	4.82 ± 1.02	5.37 ± 2.54							

^1^ Mean values of three replicates ± standard deviations. All data in each variable (either in a column or row) followed by the same letter are not significantly different by Tuckey-HSD test (α = 0.05). Letters correspond to three-way ANOVA of all data. *P*_c_, probability of cultivar; *P*_d_, probability of storage days; *P*_t_, probability of temperature; *P*_c×d_, *P*_c×t_, *P*_d×t_ and *P*_c×d×t_, probabilities of interactions. *** significant at *p* < 0.001; ** significant at *p* < 0.01; * significant at *p* < 0.05; ns, not significant.

**Table 3:** Effects of cultivar and storage temperature on peel color parameters (L*, *hue angle*, and C*) and individual carotenoid compounds (b-cryptoxanthine and lutein) after 2 days shelf life (SL) at 20 °C.

			Storage Days				Probabilities						
	Cultivar	Temperature (°C)	0 + 2	7 + 2	14 + 2	21 + 2	*P*_c_	*P*_d_	*P*_t_	*P*_c×d_	*P*_c×t_	*P*_d×t_	*P*_c×d×t_
Peel color parameters													
L*	Orange Red	1	44.03 ± 5.63 ^1^	41.96 ± 2.04	50.87 ± 1.14	45.57 ± 4.47	ns	ns	ns	ns	ns	ns	ns
		5	44.03 ± 5.63	44.69 ± 1.84	46.51 ± 4.10	49.42 ± 0.83							
	Orange Rubis	1	45.98 ± 2.22	45.66 ± 3.96	45.49 ± 4.24	45.39 ± 0.36							
		5	45.98 ± 2.22	48.14 ±2.67	45.34 ± 1.15	46.51 ± 0.36							
*h°*	Orange Red	1	49.44 ± 4.21 ab	44.04 ± 4.29 b	58.75 ± 3.38 ab	52.80 ± 6.34 ab	***	*	ns	**	ns	ns	ns
		5	49.44 ± 4.21 ab	49.85 ± 5.33 ab	52.22 ± 6.60 ab	61.34 ± 2.46 a							
	Orange Rubis	1	57.41 ± 2.46 ab	58.35 ± 9.67 ab	52.99 ± 8.88 ab	59.74 ± 6.01 a							
		5	57.41 ± 2.46 ab	63.62 ± 1.85 a	54.61 ± 1.64 ab	63.13 ± 3.49 a							
C*	Orange Red	1	39.24 ± 2.79 abc	35.21 ± 2.85 abcd	42.36 ± 1.16 a	37.63 ± 5.37 abc	***	*	ns	ns	ns	ns	ns
		5	39.24 ± 2.79 abc	36.45 ± 1.40 abcd	39.09 ± 4.78 abc	40.88 ± 1.16 ab							
	Orange Rubis	1	30.76 ± 2.26 cd	31.53 ± 3.28 cd	32.98 ± 2.20 bcd	31.31 ± 5.17 cd							
		5	30.76 ± 2.26 cd	28.49 ± 1.56 d	33.17 ± 1.56 bcd	33.94 ± 1.95 abcd							
Carotenes													
b-cryptoxanthine (μg 100 g^−1^ FW)	Orange Red	1	7.71 ± 0.25	11.62 ± 5.84	11.07 ± 2.45	10.43 ± 0.50	***	ns	ns	ns	ns	ns	ns
		5	7.71 ± 0.25	12.70 ± 1.77	8.27 ± 1.57	5.99 ± 1.80							
	Orange Rubis	1	3.48 ± 1.47	3.63 ± 0.73	3.65 ± 1.04	5.14 ± 0.53							
		5	3.48 ± 1.47	3.83 ± 0.29	3.92 ± 1.28	3.52 ± 1.33							
Lutein (μg 100 g^−1^ FW)	Orange Red	1	6.15 ± 0.76	6.39 ± 2.03	14.03 ± 3.57	6.63 ± 2.32	ns	***	ns	ns	ns	ns	ns
		5	6.15 ± 0.76	15.51 ± 0.32	13.69 ± 1.59	7.82 ± 1.82							
	Orange Rubis	1	9.95 ± 0.91	6.76 ± 2.67	13.43 ± 2.28	6.47 ± 2.29							
		5	9.95 ± 0.91	10.35 ± 4.65	16.50 ± 6.54	4.02 ± 2.19							

^1^ Mean values of three replicates ± standard deviations. All data in each variable (either in a column or row) followed by the same letter are not significantly different by Tuckey-HSD test (α = 0.05). Letters correspond to three-way ANOVA of all data. *P*_c_, probability of cultivar; *P*_d_, probability of storage days; *P*_t_, probability of temperature; *P*_c×d_, *P*_c×t_, *P*_d×t_ and *P*_c×d×t_, probabilities of interactions. *** significant at *p* < 0.001; ** significant at *p* < 0.01; * significant at *p* < 0.05; ns, not significant.

**Table 4:** Effects of cultivar and storage temperature on chilling injury (CI) incidence during cold storage (CS) and after 2 days shelf life (SL) at 20 °C.

	Cultivar	Temperature (°C)	Storage Days			Probabilities						
			14	21	28	*P*_c_	*P*_d_	*P*_t_	*P*_c×d_	*P*_c×t_	*P*_d×t_	*P*_c×d×t_
Chilling injury incidence (%)	Orange Red	1	0.00 ± 0.00 b ^1^	1.85 ± 3.21 b	5.56 ± 2.78 b	ns	***	***	*	ns	*	ns
		5	0.00 ± 0.00 b	11.11 ± 11.11 ab	19.44 ± 2.78 a							
	Orange Rubis	1	5.33 ± 9.24 b	1.33 ± 2.31 b	5.33 ± 2.31 b							
		5	4.00 ± 4.00 b	6.67 ± 6.11 b	10.67 ± 2.31 ab							
			14 + 2	21 + 2								
	Orange Red	1	0.93 ± 1.60 c	20.37 ± 1.60 ab		ns	**	ns	***	ns	ns	ns
		5	2.78 ± 2.78 bc	26.85 ± 6.99 a								
	Orange Rubis	1	12.00 ± 4.00 abc	14.67 ± 15.14 abc								
		5	12.00 ± 0.00 abc	8.00 ± 6.93 bc								

^1^ Mean values of three replicates ± standard deviations. All data in each variable (either in a column or row followed by the same letter are not significantly different by Tuckey-HSD test (α = 0.05). Letters correspond to three-way ANOVA of all data. *P*_c_, probability of cultivar; *P*_d_, probability of storage days; *P*_t_, probability of temperature; *P*_c×d_, *P*_c×t_, *P*_d×t_ and *P*_c×d×t_, probabilities of interactions. *** significant at *p* < 0.001; ** significant at *p* < 0.01; * significant at *p* < 0.05; ns, not significant.

## Data Availability

Data are contained in the article.
